# High-throughput computational design of cathode coatings for Li-ion batteries

**DOI:** 10.1038/ncomms13779

**Published:** 2016-12-14

**Authors:** Muratahan Aykol, Soo Kim, Vinay I. Hegde, David Snydacker, Zhi Lu, Shiqiang Hao, Scott Kirklin, Dane Morgan, C. Wolverton

**Affiliations:** 1 Department of Materials Science and Engineering, Northwestern University, Evanston, Illinois 60208, USA; 2 Department of Materials Science and Engineering, University of Wisconsin, Madison, Wisconsin 53706, USA

## Abstract

Cathode degradation is a key factor that limits the lifetime of Li-ion batteries. To identify functional coatings that can suppress this degradation, we present a high-throughput density functional theory based framework which consists of reaction models that describe thermodynamic and electrochemical stabilities, and acid-scavenging capabilities of materials. Screening more than 130,000 oxygen-bearing materials, we suggest physical and hydrofluoric-acid barrier coatings such as WO_3_, LiAl_5_O_8_ and ZrP_2_O_7_ and hydrofluoric-acid scavengers such as Sc_2_O_3_, Li_2_CaGeO_4_, LiBO_2_, Li_3_NbO_4_, Mg_3_(BO_3_)_2_ and Li_2_MgSiO_4_. Using a design strategy to find the thermodynamically optimal coatings for a cathode, we further present optimal hydrofluoric-acid scavengers such as Li_2_SrSiO_4_, Li_2_CaSiO_4_ and CaIn_2_O_4_ for the layered LiCoO_2_, and Li_2_GeO_3_, Li_4_NiTeO_6_ and Li_2_MnO_3_ for the spinel LiMn_2_O_4_ cathodes. These coating materials have the potential to prolong the cycle-life of Li-ion batteries and surpass the performance of common coatings based on conventional materials such as Al_2_O_3_, ZnO, MgO or ZrO_2_.

## Abstract (web summary)

Degradation of cathode materials is a key factor hindering the long-term stability of lithium ion batteries. Here, the authors develop a high-throughput computational approach to design effective cathode coating materials, proposing a selection of candidate materials to help improve cathode lifetimes.

Major intrinsic causes of cathode degradation in Li-ion batteries include instability against irreversible phase transformations^1^,^2^, for example, layered to spinel transformation in Li_*x*_MO_2_ type cathodes^3^,^4^,^5^,^6^,^7^,^8^, and dissolution of the redox-active transition metal ions into the electrolyte^7^,^9^,^10^. Corrosive species are known to attack the cathode particles and accelerate transition metal dissolution, which often leads to a significant capacity loss upon cycling^7^,^11^. Hydrofluoric acid (HF), for example, forms in the presence of only trace amount of water in the common LiPF_6_ based electrolytes^10^,^12^. A strong correlation was observed between HF content in the electrolyte and transition metal loss for common battery cathode materials including the layered LiCoO_2_, spinel LiMn_2_O_4_ and similar cathodes^7^,^9^. For LiMn_2_O_4_, in particular, disproportionation of surface Mn^3+^ to Mn^2+^ and Mn^4+^, and subsequent dissolution of Mn^2+^ into the electrolyte is triggered by the H^+^ ion (that is, acidic environments)^13^, and is a primary reason for capacity fade in this material^14^,^15^,^16^. This dissolved Mn deposits at the anode surface and further contributes to degradation^14^. Cathode–electrolyte reactions can further cause formation of a resistive solid–electrolyte interface, as a byproduct of the electrolyte breakdown^10^.

While alternative strategies such as doping^17^,^18^,^19^, tailoring the particle morphology^20^,^21^ or core–shell structures^22^,^23^,^24^ have been suggested, a common approach to suppressing cathode degradation has been applying protective coatings on cathode particles^12^,^22^,^25^,^26^,^27^,^28^,^29^,^30^,^31^,^32^,^33^. Stable binary oxides, such as Al_2_O_3_, MgO, ZnO, ZrO_2_, SiO_2_ and TiO_2_ may reduce the HF-content in the electrolyte^12^,^34^,^35^, but their performance in suppressing the transition metal-loss from the cathode or the capacity fade can vary significantly^27^,^36^. However, the complex nature of reactions between the cathode, coating and electrolyte prohibited the design of generic guidelines to find effective coatings beyond such simple binary oxides^22^,^37^. Recently, we introduced a density functional theory (DFT)-based materials design approach considering the thermodynamic aspects of binary metal oxide cathode coatings, which reproduced the known effective coatings such as Al_2_O_3_, and predicted trivalent transition metal oxides as a promising class of under-explored cathode coatings^37^. This framework was limited to only binary metal oxides, because the description of HF-reactivity and electrochemical stability of coatings were described by hypothesized reactions based on ‘chemical intuition' (that is, reactions that had predefined forms, such as M_*x*_O_1/2_+HF→M_*x*_F+½H_2_O for HF-reactivity of a metal oxide M_*x*_O_1/2_) and could not be extended to other more complex materials.

In recent years, high-throughput (HT) computational methods have significantly accelerated the search for new and better battery components^38^,^39^,^40^,^41^,^42^,^43^,^44^. Here we introduce a comprehensive HT materials design framework to discover cathode coatings by combining the Open Quantum Materials Database (OQMD)^45^,^46^, a large collection of HT DFT calculations of ∼300,000 inorganic materials, with reaction models to describe thermodynamic stability, electrochemical stability and HF-reactivity for any oxygen-bearing coating with non-intuitive, fully automated prediction of reaction products. With this framework, we design coatings with various functionalities geared towards specific battery chemistries; namely, (1) physical barriers for acid-free electrolytes, (2) HF-barriers for cathode particles fully covered with coatings and (3) HF-scavengers for particles with patchy coatings requiring active protection from HF-attack. We screen more than 130,000 oxygen-bearing materials (oxides and oxyanion compounds) available in the OQMD, and use multi-objective optimization methods, namely weighted-sum and rank aggregation, to select the best candidates for each coating category with the process outlined in Fig. 1. We further show that coatings optimized for a particular cathode material (here, for layered-LiCoO_2_ and spinel-LiMn_2_O_4_) can be designed by incorporating the cathode material itself into the chemical space; that is, considering the cathode-coating reactivity and including the cathode in all chemical reactions of the framework.

## Results

### The coating design framework

Thermodynamic and electrochemical stability are essential for a coating to ensure that the material is likely to be synthesized experimentally, and remain intact (electrochemically inactive) in the battery, respectively. On the other hand, depending on the acid content of the electrolyte and the coating morphology, different HF-related functionalities can be assigned to a given coating material, as listed in Table 1. Chen *et al*.^22^ outlined different functionalities a cathode coating may have, including physical barrier and HF-scavenger coatings. Here we consider both of these coating types, and propose a new third type as described below:

Physical barrier: in systems where HF-attack is not the dominating degradation mechanism, such as low-moisture electrolyte systems, a simple physical barrier between the cathode and electrolyte may be sufficient to suppress degradation of the cathode. Coating materials reported to serve this purpose include ZrO_2_, Al_2_O_3_, ZnO and AlPO_4_ (ref. 22).

HF-scavenger: in systems where HF is present in the electrolyte, and the coating is applied via more conventional processes where surface coverage and morphology cannot be controlled precisely, a coating material that preferentially reacts with HF can provide active cathode protection. Such HF scavenger coatings can sacrificially protect the cathode where the cathode is exposed to the electrolyte^22^. This is a common functionality expected from a cathode coating material in Li-ion batteries, with examples such as Al_2_O_3_, ZnO, MgO and Sc_2_O_3_ reported to be effective for this purpose^22^,^37^.

HF-barrier: in systems where HF is present in the electrolyte, and complete surface coverage of cathode particles can be attained during the coating process, for example, with atomic layer deposition^31^,^32^,^47^, we propose that an HF-barrier functionality can be more effective in suppressing the degradation of the cathode compared with other functionalities above. For the underlying mechanism, we hypothesize that if such a pinhole-free coating is inert to HF (that is, has a positive free energy for reacting with HF), it can retain its coverage and integrity more effectively as opposed to an HF-scavenger coating that is constantly consumed by reacting with HF.

To design a coating material with the target functionalities described above, we consider three main attributes as listed in Table 1: (i) thermodynamic stability of the coating, (ii) electrochemical stability of the coating and (iii) the reactivity of the coating with HF. Thermodynamic stability is defined as whether the material is on the convex-hull in the chemical space of elements which make up the material (not including the cathode or HF). Such stability can be readily acquired from the OQMD phase diagrams. As an example, we show the ternary Li–Ti–O phase diagram obtained from OQMD in Fig. 2a, where the stable phases such as TiO_2_, LiTi_2_O_4_, LiTiO_2_, Li_2_TiO_3_ and Li_4_TiO_4_, make up the three-phase coexistence regions, and all unstable phases decompose into a combination of such stable phases. Of the ∼130,000 oxygen-bearing materials calculated in the OQMD, ∼5,200 are thermodynamically stable against decomposition into other phases. To evaluate the attributes (ii) and (iii) we need to construct specific reactions and calculate their energies.

Electrochemical stability of a cathode coating has two components: stability under reducing (discharge) and oxidizing (charge) conditions, as illustrated schematically in Fig. 3. For an oxygen-bearing cathode coating (A_a_B_b_C_c_...)O_*x*_, a generic discharge reaction can be written as:


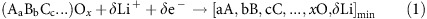


where [aA, bB, cC, ..., *x*O, *δ*Li] simply denotes the composition imposed by the reactants. The subscript ‘min' implies that the products are determined as the minimum energy combination of phases in the OQMD at this composition^48^, from which the discharge reaction energy (or the discharge potential, *E*_d_) is also subsequently calculated. Here, *δ* denotes a dilute amount, in the sense that the composition [aA, bB, cC, ..., *x*O, *δ*Li] remains within the first phase-region formed by the coating (A_a_B_b_C_c_...)O_*x*_ and other stable phases towards the Li-corner of the phase diagram. In this phase region, the Li chemical potential will be at its lowest value among all possible values along the composition path from the coating towards the Li-corner of the phase diagram, and therefore this methodology ensures obtaining the highest *E*_d_ for the given compound. This procedure of calculating *E*_d_ is illustrated in Fig. 2b with the example of the candidate Li_2_TiO_3_. Along the lithiation path of Li_2_TiO_3_, the highest lithiation voltage is attained by the reaction Li_2_TiO_3_+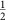
Li→
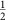
LiTiO_2_+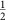
Li_4_TiO_4_ in the first phase region towards the Li corner. For a coating to be electrochemically stable with respect to reduction, the discharge potential, *E*_d_, calculated for the reaction in equation (1) needs to be low, at least lower than the discharge voltage cutoff of the cathode as shown in Fig. 3. We calculate the potential of equation (1) with respect to the Li/Li^+^ anode.

Similar to the discharge reaction, a generic charge reaction for a (A_a_B_b_C_c_...)O_*x*_ coating can be written as:


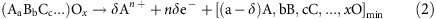


Products of this reaction, except A^*n*+^, are determined the same way as in equation (1). The ion A^*n*+^ denotes the component with the highest dissolution tendency, which we find by calculating the reaction potential for all elements (A, B, C, and so on) in the compound and choosing the highest one as the ‘charge' potential, *E*_c_. For a given element, we consider all possible oxidation states *n* with available electrochemical data (See Methods). For example, for Li_2_TiO_3_, the highest charge potential (*E*_c_) is found to correspond to the dissolution of Li^+^ via this reaction: Li_2_TiO_3_→Li^+^+e^−^+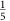
LiO_3_+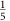
Li_4_Ti_5_O_12_. When Li is present in a compound as in this example, it usually is the element with the highest dissolution tendency, since it has one of the highest standard oxidation potentials (3.04 V) among all elements^49^. When the material does not contain Li, however, oxidation will take place via dissolution of one of the existing elements. For example, for Mn_2_VPO_7_ the highest *E*_c_ is found to correspond to the dissolution of Mn^2+^ via this reaction: Mn_2_VPO_7_→
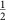
Mn^2+^+e^−^+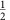
MnV_2_O_6_+MnPO_4_. For an electrochemically stable coating, *E*_c_ needs to be sufficiently negative (that is, unfavourable) that its magnitude is larger than the voltage used at the cathode charging cutoff, as shown in Fig. 3. Similar to *E*_d_, we calculate *E*_c_ with respect to the Li/Li^+^ anode. It is worth noting that the actual dissolution processes may involve multiple-ions dissolving at the same time, formation of complex ions, dissolution via self-discharge and/or multi-stage dissolution processes, and therefore equation (2) merely serves as an approximation that aims at broadly capturing the dissolution tendencies in a high-throughput fashion.

The third attribute we consider is the reactivity of the coating with HF described by the reaction:


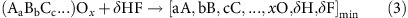


where *δ* and the products are determined with a procedure similar to the previous reactions. Again for Li_2_TiO_3_ as an example, the HF-scavenging reaction is found to be Li_2_TiO_3_+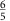
HF→
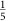
Li_4_Ti_5_O_12_+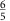
LiF+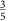
H_2_O. The measure of HF-reactivity is taken as the magnitude of the free energy of the HF-scavenging reaction, *G*_s−HF_.

Finally, for HF-scavenger coatings, we should note that equation (3) will produce fluorides, which are likely to have discharge potentials higher than the oxide coating itself 37. Therefore, for HF-scavenger coating design, we further include the discharge potential of the fluorinated reaction products (*E*_d_(products)) as a criterion, which is basically the potential calculated by replacing (A_a_B_b_C_c_...)O_*x*_ in equation (1) with the mixture [aA, bB, cC, ..., *x*O, *δ*H, *δ*F]_min_ obtained as the products of the reaction in equation (3). As an example, for an Li_2_TiO_3_ coating, this reaction is found to be Li_4_Ti_5_O_12_+(6LiF+3H_2_O)+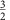
Li→2Li_2_TiO_3_+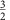
LiTi_2_O_4_+(6LiF+3H_2_O). In this particular example, the species in the parenthesis, LiF and H_2_O, do not participate in the reaction and remain intact. As we showed throughout this section with examples, reactions become complex and non-intuitive even for ternary candidate coatings, and the whole procedure described here is fully-automated.

### Multi-objective optimization for material selection

**Figure 1:**
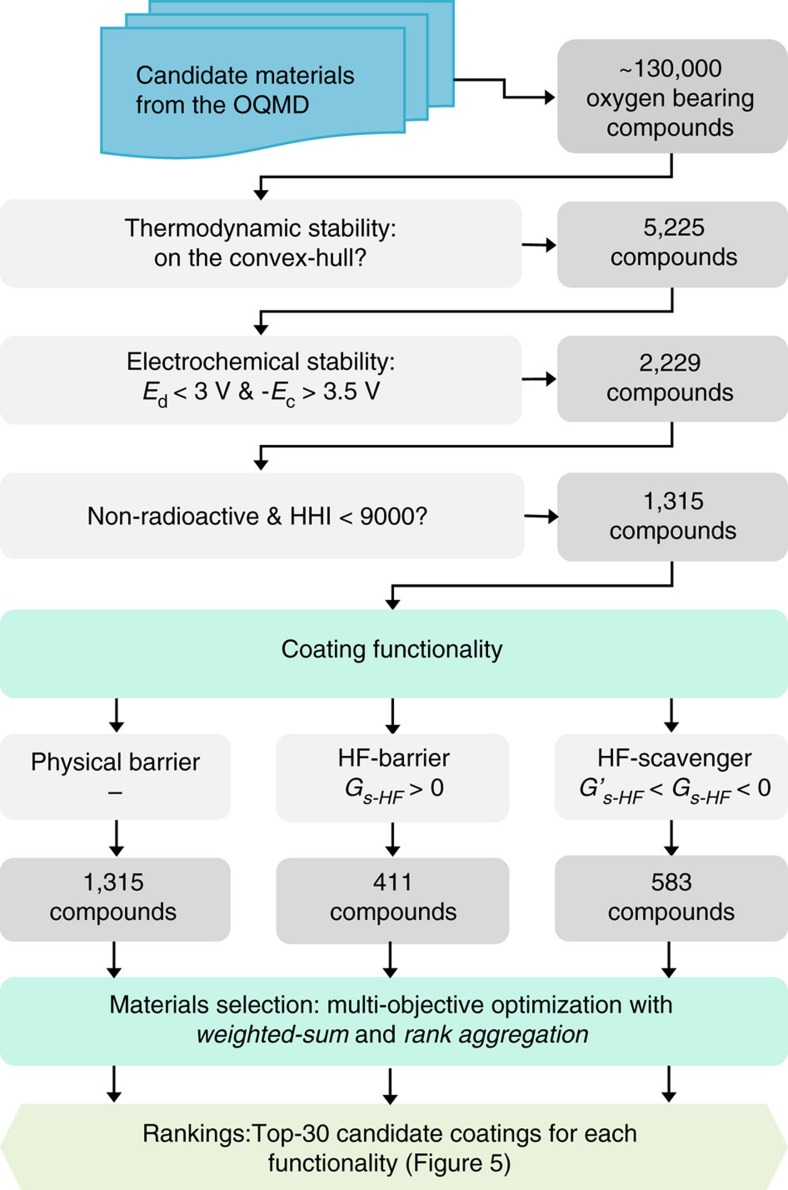
The flowchart of the HT coating design framework. The filters applied on each design attribute are listed along with the number of compounds that pass each filter. 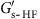 is the excess reactivity limit.

**Figure 2:**
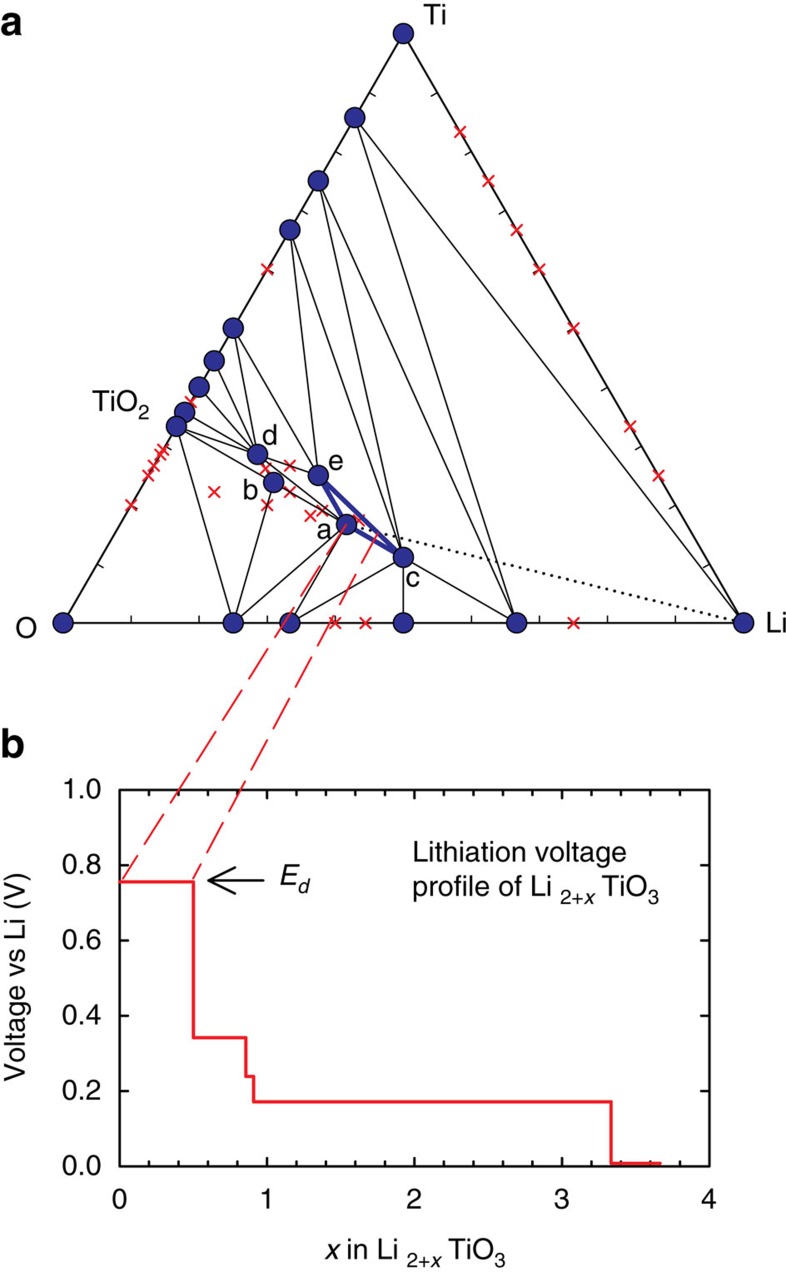
Stability and lithiation of Li_2_TiO_3_ coating. (**a**) Ternary Li–Ti–O phase diagram obtained from the OQMD, where the stable and unstable phases are shown as circles and crosses, respectively. Compounds marked as a,b,c,d,e correspond to Li_2_TiO_3_, Li_4_Ti_5_O_12_, Li_4_TiO_4_, LiTi_2_O_4_ and LiTiO_2_, respectively. The O_2_ chemical potential corresponds to *T*=298 K and *P*=1 atm. Dotted-line shows the lithiation path of Li_2_TiO_3_, and the first phase region along this path is highlighted with bold tie-lines. The corresponding voltage profile of Li_2+*x*_TiO_3_ as a function of *x* along this path is given in **b**, where the arrow points at the highest voltage step; that is, the discharge potential criterion (*E*_d_) of this coating candidate as we use in the framework.

**Figure 3:**
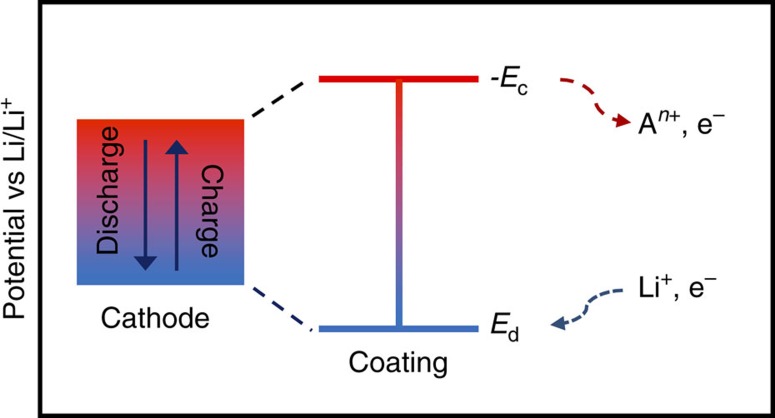
Electrochemical stability window of the coating. The schematic compares the operating voltage window of a cathode to charge and discharge potentials (that is, *E*_c_ and *E*_d_, respectively) of an electrochemically stable coating.

The application of the HT coating design framework involves a series of material screening and selection steps as shown in Fig. 1. We first filter the oxygen-bearing candidate materials obtained from the OQMD on the basis of their thermodynamic and electrochemical stabilities, as well as HHI values and radioactivities. Following the application of this initial set of filters, the remaining materials are screened separately for the three main categories of coating functionalities (physical barrier, HF-barrier and HF-scavenger) using filters relevant for each functionality. Therefore, this step yields different pools of candidate materials for each category. In the next step, we further apply multi-objective optimization methods to rank the materials and predict the top candidates for each coating functionality. Below we discuss the results of these steps in the coating design workflow in detail.

**Figure 4:**
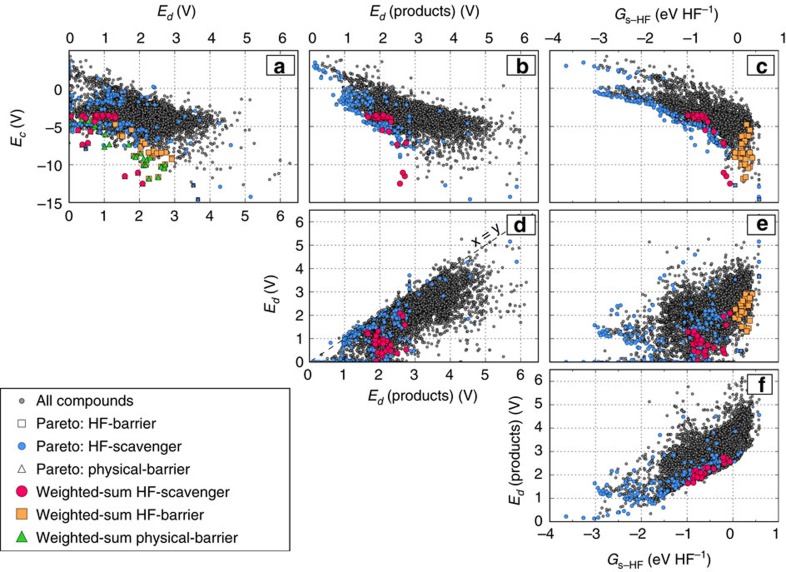
Matrix plot of attributes employed within the coating design framework for pair-wise comparison. Panels **a**–**f** represent each unique attribute pair. Based on the ideal trends of attributes for different types of coatings listed in Table 1, materials on the Pareto-front for each coating type are determined. Top 30 coating materials found by weighted-sum method are also shown for each coating category.

We present the calculated coating design attributes in Fig. 4 as a matrix plot for the ∼5,200 thermodynamically stable oxides and oxyanion compounds in the OQMD (values of attributes are provided in Supplementary Data 1). For physical barrier coatings, the only relevant panel is (a) *E*_c_ versus *E*_d_, for HF-barrier coatings the relevant panels are (a) *E*_c_ versus *E*_d_, (c) *E*_c_ versus *G*_s−HF_ and (e) *E*_d_ versus *G*_s−HF_, and for HF-scavenger coatings, all panels are relevant. For all these coating categories, the lower values are optimal for all attributes, except the *G*_s−HF_ of HF-barrier, which by definition (Table 1) needs to be high. *E*_c_ versus *E*_d_ is the key panel that describes the electrochemical stability for all types of coatings. Since both *E*_c_ and *E*_d_ should be low for an electrochemically stable material, a candidate with the ideal combination of these attributes should be towards the lower left corner of the panel. However, there is roughly an opposite trend between *E*_c_ and *E*_d_; that is, an improvement (decrease) in *E*_d_ often leads to an increase in *E*_c_ of a candidate coating. Therefore *E*_c_ and *E*_d_ are objectives that are mostly conflicting.

Similar conflicting trends also exist among other attributes. For HF-scavenger coatings, attribute pairs in all three panels in the first row in fact show conflicting trends in Fig. 4. As expected from the higher electronegativity of fluorine compared with other anion forming elements, products of HF-scavenging reactions (that is, mixture of fluorinated metal oxides) clearly react more favourably with Li than the oxides themselves and yield higher lithiation potentials as evident from Fig. 4d, consistent with the findings in Aykol *et al*.^37^ Plots of *E*_d_ against *E*_d_(products), as well as *E*_d_ (or *E*_d_(products)) against *G*_s−HF_ show non-conflicting trends; that is, materials with ideal attributes can be located at the lower left corner of each of these plots. These materials, however, clearly exhibit the highest *E*_c_ values, and therefore cannot make promising candidates.

Due to the conflicting nature of attributes in the multi-objective optimization (MOOP) for material selection, and highly scattered data with no clear structure, it is not possible to find the best coating materials simply by a pair-wise comparison of attributes. Thus, we now apply a preliminary screening of attributes as described in Methods, followed by application of two material selection methods; weighted-sum and rank aggregation to tackle this MOOP. For the ∼5,200 thermodynamically stable candidates, screening reduces the number of physical barrier, HF-barrier, and HF-scavenger coating candidates to 1,315, 411 and 583, respectively, which can now be further ranked using weighted-sum and rank aggregation methods to find the best candidates.

**Figure 5:**
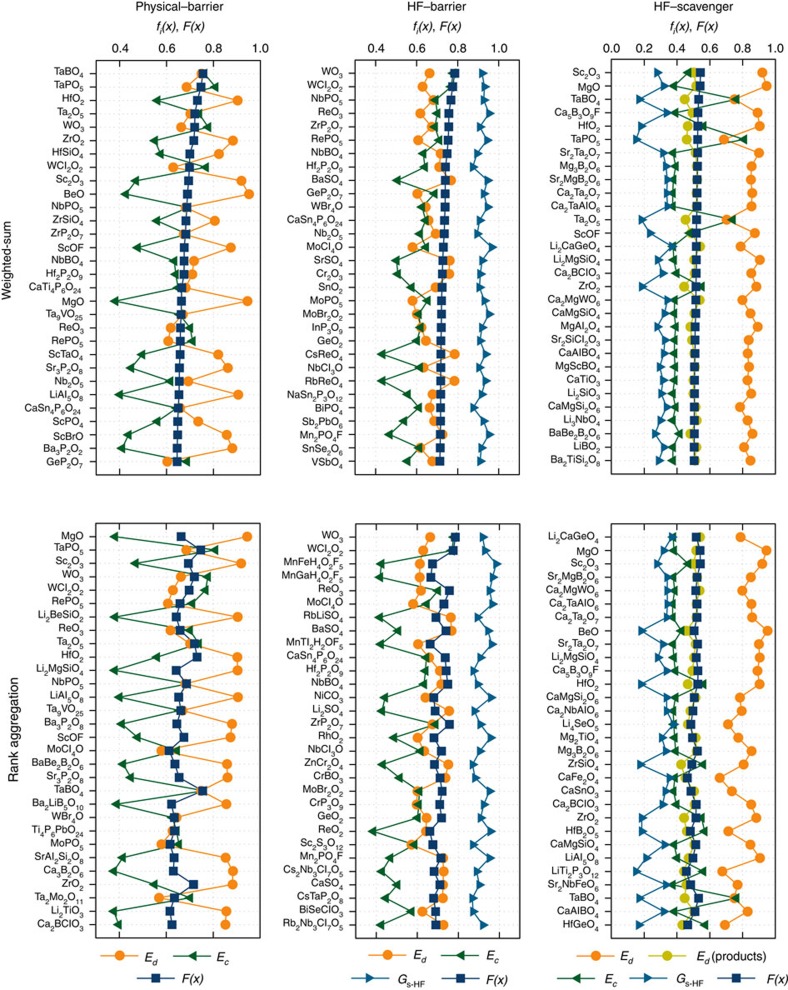
Material rankings for functional coatings. The rankings for the top 30 candidates obtained with weighted-sum and rank aggregation methods for physical barrier, HF-barrier and HF-scavenger coatings are shown. Individual attributes, *f*_*i*_(*x*) and the resulting global objective *F*(*x*) are given for each material. Individual attributes and global objectives are also given in the rank aggregation panels, to allow comparisons between each method. The highest-rank material is on top in each list. Note *f*(*x*) of *G*_s−HF_ denotes opposite ideal limits for HF-barrier and HF-scavengers as defined in Table 1.

In Fig. 5, for each coating type, we show the rankings of top 30 (an extended list of rankings can be found as Supplementary Data 2) candidates obtained with the weighted-sum and rank aggregation methods, along with the global objectives (*F*(*x*)), and normalized attributes (*f*(*x*)) of materials. For a given coating type, while the absolute ranks obtained with weighted-sum and rank aggregation are not necessarily identical, there is significant overlap between the lists obtained with these different methods. The *f*(*x*) and *F*(*x*) of top materials in rank aggregation lists are also found to be similar to those in the weighted-sum lists. Therefore, as two characteristically different MOOP methods yield similar results, we conclude that our predictions of the final top candidates do not depend strongly on the material selection method, and represent a viable set of solutions for the MOOP. The selected candidates we recommend below appear in the top-list with both methods.

### Deterministic material selection for HF-scavenger coatings

The trade-offs between conflicting objectives in MOOP of designing cathode coatings do not allow selection of a single ‘ultimate' coating, and therefore we obtain lists of optimal candidates in Fig. 5. If the relative importance of attributes (*w*_*i*_ in *F*(*x*)) were known precisely, one would be able to solve the multi-objective cathode coating design problem within our model exactly. While such high-level information is not available, we know that the goal in designing HF-scavenger coatings is to protect the cathode from acid-attack, while remaining electrochemically inactive. These requirements can be tested with chemical reactions defined in equations (1, 2, 3) by adding the additional constraint that the coating does not react with a specific cathode of interest. Therefore, in analogy with previous work on the stability of electrode–electrolyte pairs^50^,^51^,^52^, we now consider this additional constraint by studying what the phase mixture would be for any cathode+coating pair, which can be expressed in the form of a reaction as:


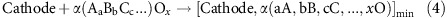


Here *α* denotes a dilute amount, analogous to the definition of *δ* in equation(1, 2, 3), (A_a_B_b_C_c_...)O_*x*_ represents the coating and products are again found as the lowest energy combination of the phases at the given composition in the OQMD chemical space. As an example, below we show the reaction predicted to take place between the common LiCoO_2_ cathode and an Al_2_O_3_ coating:


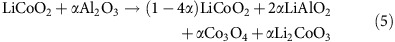


This reaction indicates that Al_2_O_3_ will react with LiCoO_2_, reduce the amount of this active cathode, and lead to precipitation of LiAlO_2_ and other Co-oxides. When Al was added to LiCoO_2_ in experiments, a layered LiAlO_2_−LiCoO_2_ solid solution was found to form^17^. Detailed investigations of Al_2_O_3_-coated LiCoO_2_ and similar layered cathodes also revealed similar solid-solutions near the cathode surface^53^,^54^. Therefore, as the end-member of this solid-solution, prediction of the stability of LiAlO_2_ with LiCoO_2_ as in equation (5) is consistent with these experiments within the limits of our bulk thermodynamic models based on the OQMD phases.

The products of equation (4); that is, [Cathode, *α*(aA, bB, cC, ..., *x*O)]_min_ can be substituted for (A_a_B_b_C_c_...)O_*x*_ on the reactants side of reactions in equations (1, 2, 3) to keep account of the amounts of cathode and coating materials that go through the reactions. For example, HF-attack reaction for the equilibrium mixture of LiCoO_2_ cathode-*α*Al_2_O_3_ coating described above is found to be:


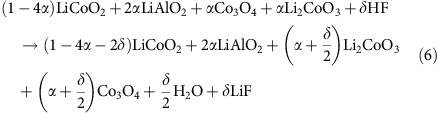


This reaction demonstrates that under HF-attack, LiCoO_2_ is consumed while the amount of LiAlO_2_ remains the same, indicating the nominal Al_2_O_3_ coating, when allowed to fully react with LiCoO_2_ to form the equilibrium phase mixture in equation (5), will not provide an HF-scavenging protection for LiCoO_2_. This result is counter-intuitive, as pristine Al_2_O_3_ is a strong HF-scavenger^22^,^37^.

Here we select two of the most common cathodes that represent the layered and spinel families; that is, LiCoO_2_ and LiMn_2_O_4_, and search for optimized HF-scavenger coating materials among the ∼5,200 thermodynamically stable candidates using the approach outlined here. To summarize, we first substitute the equilibrium cathode+coating phase mixture [Cathode, *α*(aA, bB, cC, ..., *x*O)]_min_ for (A_a_B_b_C_c_...)O_*x*_ in equations (1, 2, 3), then find the reaction products and energies, and evaluate the following criteria as pass/fail filters: (i) coating and cathode are stable together (they do not react to form other phases), (ii) cathode is protected (not consumed) in HF-attack reaction in equation (3) and (iii) coating does not participate in the electrochemical activity upon charge and discharge in reactions equations (1 and 2). Since we can keep track of the amount of cathode and coating materials in reactions, these pass/fail filters eliminate the need for most of the screens required in MOOP, and we only apply the HHI, radioactivity and excessive-reactivity screens described in Methods.

While this framework can yield optimal coatings for a given cathode without MOOP, it does not allow ranking the candidates as in MOOP, so we also consider ‘nearly optimal' coatings not to miss good candidates because of inherent uncertainties in calculated free energies. For example, in the charge reaction, we assume a dilute concentration in electrolyte for the dissolving ion in equation (2) (See Methods), and use the standard oxidation potentials from aqueous solutions, both of which are approximations to actual battery systems. We therefore expect the uncertainty in calculated *E*_c_ values to be larger than that in other calculated thermodynamic quantities. For this reason, we relax the *E*_c_ filter by an amount equivalent to ∼2 orders of magnitude deviation in ion activity in the electrolyte, which corresponds to an approximately ±0.12 V window in the potential of a one-electron reaction (approximated using the 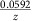
log(*K*) term in the Nernst equation at 25 °C). We use this value as a buffer in *E*_c_ to find the nearly optimal candidates. In other words, reactions with *E*_c_ values up to 0.12 V higher than that of the cathode material are still allowed to pass the electrochemical stability filter.

The matrix plots of cathode+coating systems (Supplementary Figs 1 and 2) are not similar to Fig. 4 because the chemical reaction spaces are now systematically bounded by reactions pertaining to the cathode material itself (Supplementary Table 1). For this reason, in contrast to MOOP in Fig. 4, the ideal points in plots (that is, lower left corners), are now well-defined in every panel of matrix plots in Supplementary Figs 1 and 2. We found that *E*_d_(products) is an attribute that conflicts with *E*_c_, and there is no material that would satisfy both of the criteria we set above for these quantities as a coating on LiCoO_2_ or LiMn_2_O_4_. Since *E*_d_ and *E*_c_ are essential to define the electrochemical stability of the coating material, and the amount of HF-scavenging reaction products will presumably be much less than the coating material, we exclude the lithiation of these reaction products (that is, *E*_d_(products)) from this particular analysis.

The results of this design approach where the cathode is in the chemical space along with the coating are shown in Table 2. Out of 5,225 thermodynamically stable oxide and oxyanion compound candidates, 1,792 are stable (had a tie-line) with LiCoO_2_ and 1,237 candidates can potentially protect LiCoO_2_ from HF-attack. Of these, 405 compounds provide both cathode+coating stability and protection from HF, while only a few are electrochemically stable (that is, inactive) and are listed in Table 2. For LiMn_2_O_4_, number of compounds stable with it is 1,003, and number of those that protect the cathode from HF-attack is 2,841. Surprisingly, the number of compounds that satisfy both of these filters is only 81, among which only the ones listed in Table 2 are electrochemically stable.

## Discussion

In MOOP, the physical barrier category is dominated by oxides, phosphates and a few borates of early d-block metals such as Ta, W, Hf, Zr, Nb and Sc in Fig. 5. Among the oxides, HfO_2_, Ta_2_O_5_, WO_3_, ZrO_2_, Sc_2_O_3_, MgO and LiAl_5_O_8_; among the phosphates, TaPO_5_ and NbPO_5_; and among the borates, TaBO_4_ appear as promising physical barrier coatings in both weighted-sum and rank aggregation lists. The only additional attribute considered for the HF-barrier coatings compared with physical barriers is low HF reactivity (that is, positive *G*_s−HF_), and therefore there is some overlap, especially among the metal-phosphates, between those ranked lists in Fig. 5. In addition to WO_3_, important candidates for the HF-barrier coating category include NbBO_4_, BaSO_4_ ZrP_2_O_7_, Hf_2_P_2_O_9_, Mn_2_PO_4_F and CaSn_4_(PO_4_)_6_. Overall, 4*d* and 5*d* metal phosphates emerge as a new class of promising physical- and HF-barrier coatings.

For the HF-scavenger category, while known effective HF-scavenger coatings such as MgO, ZrO_2_ and the previously predicted Sc_2_O_3_ (ref. 37) are in the top-lists, the rest of the promising compounds span an unexpectedly wide range of chemistries including oxides, borates, fluoroborates, oxyfluorides, chlorates and silicates of Li, Mg, Ca and Sr. Many of these compounds have a Ta, W, Ti, Nb or Al component as well. Most of the HF-scavenger coating materials ranked in top-30 lists by both ranking methods, such as Ca_5_(BO_3_)_3_F, Mg_3_(BO_3_)_2_, Sr_2_Mg(BO_3_)_2_, TaBO_4_, CaAlBO_4_, Li_2_MgSiO_4_, CaMgSiO_4_, CaMgSi_2_O_6_, Ca_2_Ta_2_O_7_, Ca_2_TaAlO_6_, Ca_2_ClBO_3_, Ca_2_MgWO_6_ Li_2_CaGeO_4_ and SrTa_2_O_7_, or those listed in Table 2 are unprecedented candidates that would pave the way for classes of HF-scavenger coating materials beyond binary metal oxides. Some of the Li-bearing compounds such as Li_2_SiO_3_, Li_3_NbO_4_, LiBO_2_, Li_4_SeO_5_ and LiAl_5_O_8_, appear only in either of the weighted-sum or rank-aggregation lists, but they are still worth highlighting since such compounds are more likely to be good Li-ion conductors. A common feature among almost all HF-scavenger coatings is that they contain at least one of the s-block elements Li, Sr, Mg, Ca and Ba, both in Fig. 5 and in Table 2. These elements form oxygen-bearing compounds that vigorously react with HF^37^, and have only one stable oxidation state so their compounds are often electrochemically stable. The reaction free energy of LiCoO_2_ with HF is more negative than that of about 70% of candidate coatings considered, whereas for LiMn_2_O_4_ the same number is about 50%. Therefore only materials that very strongly react with HF such as the s-block containing metal oxides/oxyanion compounds are capable of protecting LiCoO_2_, while compounds bearing *p*- and *d*-block elements along with *s*-block are present among optimal coatings for LiMn_2_O_4_, as listed in Table 2.

Among the coatings that have been widely tested experimentally, and often found to yield sufficient protection^12^,^22^,^25^,^26^,^27^,^28^,^29^,^30^,^31^,^32^,^33^,^37^, MgO, ZrO_2_, and Ta_2_O_5_ are in the top 30 HF-scavenger lists in Fig. 5. Other coating materials that are well-known to be effective HF-scavengers, namely Al_2_O_3_, TiO_2_ and ZnO also passed all screens and are predicted to be effective, only with relatively lower rankings at 128th, 139th and 313th, respectively, among the weighted-sum-ranked HF-scavengers of ∼130,000 candidates (that is, considering the size of the candidate pool, these are still predicted to be near top of the list). Similarly, we find that AlPO_4_, a well-known effective physical barrier type of coating^29^, is relatively highly ranked (68th) in the weighted-sum physical barrier list, out of ∼130,000 candidates, and therefore the physical barriers predicted in Fig. 5 are likely to perform at least as good based on our thermodynamic analysis. Thus, the above-mentioned findings validate the predictive capability of our framework by locating the already-known effective coatings, and indicate that our higher ranked, yet unexplored predictions are likely to perform at least as good, or even better than these already known coatings.

**Figure 6:**
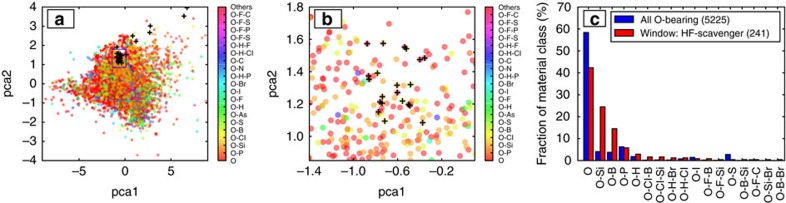
Statistical analysis of effectiveness of material classes for HF-scavenging. (**a**) Principal component analysis (PCA) for HF-scavenger coating attributes for the ∼5,200 thermodynamically stable oxides. The promising HF-scavengers listed in Fig. 5 are marked with a ‘+' sign, and their approximate region in the plot is highlighted by a rectangular window. This highlighted window is magnified in **b**. In **c**, we compare the distribution of material classes among all stable O-bearing compounds to that of all promising HF-scavengers from **b**. O-bearing compounds are exclusively classified on the basis of the (poly)-anion forming elements they have in addition to oxygen; namely, P, Si, Cl, B, S, As, H, F, I, Br, N and C. For example, compounds labeled as O do not include those labeled as O–P, and similarly O–H–P or O–F–P are not subsets of the O–P category.

The variation of weighted-sum *F*(*x*) within the top 30 HF-scavengers list in Fig. 5 is relatively slow, which results in many similarly good candidates not appearing in the list. But from a practical point of view, rather than extending the list and suggesting more candidates, we can carry out a broader analysis to find out which material classes are in general more promising than the others as HF-scavengers. For instance, are plain oxides the best HF-scavengers? To do this analysis, we first reduce the dimensionality of the 4-D HF-scavenger design space of *E*_c_, *E*_d_, *E*_d_(products), *G*_s−HF_ to a two-dimensional space via principal component analysis (PCA)^55^. The resulting two highest variance principal components, pca1 and pca2, are shown in Fig. 6a. These components respectively explain 78 and 16% (or in total 94%) of the overall variance of the entire four-dimensional data set. There are no clearly separated clusters of different material classes in Fig. 6a. However, we see that except for a few outliers, promising compounds predicted with weighted-sum are clustered around the same region as shown in detail in Fig. 6b. Materials in proximity of these weighted-sum predicted HF-scavengers are also likely to be good candidates. We analyse the distribution of material classes among the 241 candidates in this window, and compare it to the overall distribution of 5,225 stable O-bearing compounds in Fig. 6c. While plain oxides, that is, O-bearing compounds with no other polyanion forming elements, have the largest fraction among the stable compounds, their fraction is smaller among the promising HF-scavengers list. On the other hand, compared with plain oxides or other oxygen polyanion material classes, the fraction of silicates and borates in the promising HF-scavengers window is 5–10 times larger than their fraction among all stable O-bearing compounds. In other words, there is a much higher chance of discovering effective HF-scavengers in these two material classes, compared with the others. Thus, we recommend future experimental efforts which aim to explore coatings with HF-scavenging functionality beyond plain oxides to focus on silicates and borates. In fact, recent experiments on borate coatings corroborate these findings^56^.

To understand why borates and silicates yield more effective coatings than oxides, we compare the distribution of the four main thermodynamic attributes of HF-scavengers in the framework in Supplementary Fig. 3. Among oxides, borates and silicates, there are no significant variations in the distribution of HF-scavenging tendencies, charge potentials, or discharge potentials of fluorinated HF-reaction products. On the other hand, the fraction of borates and silicates with lower discharge potentials is considerably larger than oxides. Given the similarity of the rest of the properties, the relatively stronger stability against reduction increases the likelihood of borates and silicates of resulting in more effective HF-scavenger candidates compared with plain oxides.

Promising coatings predicted with MOOP in Fig. 5 and those optimized for specific cathodes in Table 2 are not necessarily similar materials. The different promising coatings can be applied effectively by considering the differences in the design approaches with which they were predicted, and connecting that to the actual cathode-coating morphology. In particular, for a system where the coating is thick enough such that the bulk of the coating away from the cathode-coating interface preserves the targeted nominal coating composition, a model where cathode is not considered in the chemical space maybe more applicable; therefore, the promising coatings listed in Fig. 5 are geared more towards such morphologies. For a system where the coating is thin and/or the coating process allows the reaction of the bulk of the coating material with the cathode, a model where cathode is present in the chemical space is most applicable, and therefore the predictions in Table 2 are most suitable.

Thermodynamic properties of coatings as included in this framework are necessary but not sufficient to find optimal coatings. One aspect of cathode-coating reactivity we did not include in the deterministic material selection procedure is how this reactivity might change upon delithiation^57^. Such an assessment of cathode-coating reactivity is hindered by the fact that cathodes themselves become unstable against decomposition into other phases under such conditions^8^,^57^, and therefore the ground state thermodynamic mixture would not include even the cathode itself. Experiments show the decomposition of common cathodes (for example, layered to spinel transformation in Li_*x*_CoO_2_)^3^ does not take place spontaneously, but often is a relatively slow degradation mechanism. Thus, we assume the reactivity of the coating with the delithiated cathode is likely to take place relatively slowly, compared with the synthesis/heat treatment of fully-lithated cathode/coating particles, where the reactions are expected to be more spontaneous and therefore more critical to consider in our framework.

In addition to reactivity with delithiated cathodes, further reactivity of the coating with other components of the battery, and in particular with the organic electrolyte could not be addressed directly within this high-throughput framework. To partially address this, we use a filter of ‘excess reactivity' in HF scavenger reactions (see Methods), but the actual reactivity with electrolyte is not necessarily captured for many systems. For example, compounds containing hydrogen (for example, SrHClO, SrHBrO, and so on in Table 2) may further trigger acid production by supplying protons to the electrolyte, but we did not exclude such H-bearing compounds from our analysis, as previous work shows metal hydroxides can still be as effective as corresponding oxides^58^.

Electronic and Li-ion conductivity are the two other important factors in coating design as addressed in recent studies^59^,^60^, both of which should be high enough to have an effective coating material that does not result in large interfacial impedance and improves the electrochemical performance of the battery. These properties, however, strongly depend on extrinsic factors such as the morphology, microstructure and synthesis conditions, and therefore are hard to model and show significant variations. For example, Xu *et al*.^60^ showed that most of the common coating materials do not allow adequate Li ion transport in crystalline form whereas their amorphous counterparts often provide fast enough Li^+^ diffusion that allows effective conformal coating morphologies^59^,^60^. Furthermore, ultra-thin atomic layer deposition coatings can actually supply sufficient electron and ion conduction even if the bulk counterpart cannot^22^,^47^. If it becomes available in the future, Li-ion diffusivity data can easily be added as a screen/attribute for ranking materials within the same framework. As the diffusion calculations are computationally expensive, our screening strategy will reduce the number of compounds for which these computations would need to be done.

In this work, we have developed a high-throughput thermochemical framework to design cathode coating materials for Li-ion batteries. The framework includes model reactions to describe the thermodynamic stabilities, electrochemical stabilities (both at charge and discharge), and HF-reactivities of candidate oxides and oxyanion compounds. Thermodynamic stability is evaluated by finding whether a compound decomposes into other phases available in the OQMD. Electrochemical stability is evaluated based on the reactivity of the coating with Li^+^ during discharge, and dissolution of the coating components into electrolyte during charge of the battery. To calculate the free energies of these reactions at room temperature, DFT energies of materials from the OQMD are used in conjunction with experimental thermochemical/electrochemical data including entropy contributions to gaseous reference states, standard reduction potentials of elements, and the Nernst relation. Reaction products are found in a fully-automated fashion as the lowest energy combination of phases in the OQMD at the composition of the reactants. We have applied the framework to screen more than ∼130,000 oxygen-bearing materials available in the OQMD and predict coatings with various functionalities: physical barrier, HF-barrier and HF-scavenger. To select the best candidates for each coating category, we used weighted-sum and rank aggregation multi-objective optimization methods. The promising physical and HF-barrier coatings we find include metal oxides and phosphates such as WO_3_, LiAl_5_O_8_, ZrP_2_O_7_, Hf_2_P_2_O_9_, TaPO_5_, CaSn_4_(PO_4_)_6_ and promising HF-scavenger coatings include hitherto unexplored candidates such as Li_2_CaGeO_4_, LiAl_5_O_8_, TaBO_4_, LiBO_2_, Mg_3_(BO_3_)_2_, Ca_5_(BO_3_)_3_F, Ca_2_Ta_2_O_7_ and Li_2_MgSiO_4_, to name several examples that would be the most useful for subsequent experimental testing. With the aid of principal component analysis, we identified silicates and borates as the two material classes that provide a higher probability to yield effective HF-scavengers compared with all other oxygen-bearing material classes. In addition to multi-objective optimization, we presented a deterministic materials design approach to find cathode-specific coatings by including the cathode in the chemical space, evaluating stability of the cathode-coating pair, and allowing the cathode to participate in the chemical reactions of the framework along with the coating material itself. The optimal coatings for a given cathode are then found by determining if the cathode remains intact when the coating is applied or when attacked by HF, and if the coating interferes with the electrochemical activity of the cathode. With this design strategy, we found promising optimal coatings such as Li_2_SrSiO_4_, Li_2_CaSiO_4_ and CaIn_2_O_4_ coatings for LiCoO_2_ and Li_2_GeO_3_, Li_2_TiSiO_5_, Li_4_NiTeO_6_, Ca_2_Mn_3_O_8_ and Li_2_MnO_3_ for LiMn_2_O_4_. The functional coatings predicted in this work provide both a qualitative mapping of where to find promising compounds and an extensive list of promising compounds for experimental testing.

## Methods

### Methodology of calculating reaction free energies

The HT materials design framework presented here relies on the availability of accurate formation energies to calculate compound stabilities and reaction energies. Accurate and efficient computation of formation energies of inorganic materials using DFT in a HT fashion is an ongoing pursuit^41^,^46^,^61^,^62^. Current methodologies, such as the OQMD^45^,^46^ and Materials Project^41^ combine DFT, DFT+*U* with optimal *U* parameters^63^,^64^,^65^, and chemical potential corrections to mitigate systematic errors and bridge DFT and DFT+*U* calculations^64^,^65^,^66^,^67^,^68^,^69^. For all solid phases, we use the formation energies of materials available in the OQMD, which were calculated using the Vienna Ab-initio Simulation Package^70^,^71^. The OQMD currently includes nearly all unique and calculable compounds available in the Inorganic Crystal Structure Database (ICSD), together with a growing number of hypothetical compounds derived from decorations of commonly occurring crystal structures. The hypothetical compounds play a key role in the OQMD, as they populate chemical spaces which may not have been explored adequately in experiments and lack information on compounds and their structures (for example, chemical spaces bearing H- and F- along with less common elements). Therefore, such compounds help provide a better approximation to the energetics of the convex-hull compared with only including ICSD phases, and yield more accurate reaction energies in such under-explored chemical spaces. Further details of the DFT settings, procedure of fitted elemental reference states and sources of OQMD structures can be found in Kirklin *et al*.^46^

Here, we describe our approach to calculating the free energies of reactions by combining DFT formation energies in the OQMD, experimental thermochemical data for gaseous species and experimental electrochemical data for solvated ions, which requires using consistent reference energies. Our goal is to combine thermochemical information from these different sources in a coherent HT framework to describe reaction energies at around room temperature, in a framework similar to the Pourbaix diagram formalism outlined by Persson *et al*.^72^ The standard free energy of a reaction is obtained as,


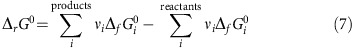


where *ν*_*i*_ and Δ_*f*_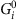 denote the stoichiometric coefficient the species *i* takes in the reaction, and its standard free energy of formation, respectively. For solid compounds, Δ_*f*_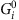 can be written as,


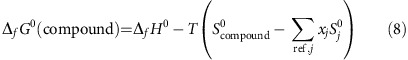


where *j* denotes the elemental reference states, *x* denotes the amount of element *j* in the compound, and *H* and *S* denote enthalpy and entropy, respectively. For solid phases, we assume *pV* contributions are negligible, and approximate Δ_*f*_*H*^0^ using DFT formation energies in the OQMD. Besides, since the change in Δ_*f*_*H* between 0 and 298 K is often on the order of a few meVs per atom^65^, we approximate the enthalpy of formation of solid phases at room temperature as Δ_*f*_*H*^0^[298 K]≈Δ_*f*_*H*^OQMD^[0 K].

To obtain the free energies of formation at room temperature for the compounds available in the OQMD, we need to include the entropy contributions in equation (8). Near room temperature, for solids we assume 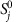
≈0 (including the compound and solid elemental references) compared with that of the gaseous reference states. Thus, within our framework, Δ_*f*_*G*^0^ of a compound can be written as,


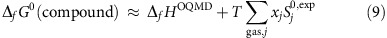


Here, for the gaseous reference states (O_2_, F_2_, Cl_2_, H_2_ and N_2_), the 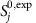 at room temperature are obtained from the JANAF tables^73^. While the vibrational zero-point energies, for example, of atoms such as hydrogen in hydrogen bearing compounds may be non-negligible, we assume that an averaged zero-point energy is included in the OQMD formation energies as the corresponding chemical potential corrections of these gaseous reference states (including H_2_) were fitted to experimental data^46^. The room temperature free energy of formation of liquid H_2_O and dilute HF in electrolyte (Δ_*f* _*G*^0^(H_2_O) and Δ_*f* _*G*^0^(HF), respectively) are further approximated using the experimental thermochemical data^49^. Further details of implementing experimental thermochemical data for the phases described above into our framework can be found as Supplementary Table 2.

Since equation (9) allows us to calculate the room temperature Δ_*f* _*G*^0^ of any arbitrary compound in the OQMD, we can use these OQMD-derived free energies along with the experimental free energies, such as of solvated ions. For the standard free energy of formation of a solvated ion, Δ_*f* _*G*^0^(ion)^*n*+^, we use the experimental standard oxidation potential of the ion *ɛ*(ion)^*n*+^:


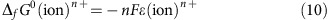


where *F* is the Faraday's constant and *n* is the valence state of the ion in solution. *ɛ*(ion)^*n*+^ (with respect to standard hydrogen electrode) are obtained from the National Bureau of Standards tables^49^.

For elemental reference states we have by definition,


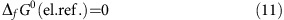


Using Δ_*r*_*G*^0^ and Δ_*f*_*G*^0^ defined above, free energy of a given reaction can be calculated as,


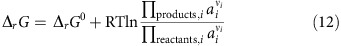


The activities of solid phases are one within our level of approximation. Since the electrolyte is Li-based, Li^+^ activity is assumed unity, whereas for the other dissolving ions, we assume a small activity of *a*_ion≠Li_=10^−6^ to approximate the actual dilute concentrations in the electrolyte. Half or full cell potentials can further be obtained using the Nernst relation as,


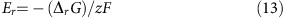


where *z* is the number of electrons transferred in the reaction. We use the Li/Li^+^ as the reference for the voltages. For equation (2), further deposition of the dissolved ions at the anode is also possible, but not considered in this work.

### Materials selection

Selecting the best candidates among thousands of materials based on multiple thermodynamic attributes described is a MOOP, which yields many non-dominated (Pareto-optimal) solutions^74^. Here we implement multiple strategies to select materials for target coating applications: (i) an initial screening to narrow down the search space, (ii) followed by two alternative procedures to address the MOOP: global weighted-sum objective and rank aggregation.

We apply a series of preliminary screens before solving the MOOP to filter out materials that do not pass our basic thermodynamic, electrochemical, and reactivity requirements, as listed in Table 1. Thermodynamic stability, in fact, is a screen which reduces the number of candidate oxygen-bearing materials from ∼130,000 to ∼5,200, and significantly narrows down the search space. For HF-scavenger coatings, we apply a screen of *G*_s−HF_<0, to ensure the coating provides the scavenging functionality. We further set the lower limit for *G*_s−HF_ as that of the basic oxide CaO as done previously^37^, to eliminate materials with ‘excessive reactivity' against other components of the battery. For HF-barrier coatings, we adopt a screen of *G*_s−HF_>0, so as not to allow this type of coatings to react with HF. For all coatings, we select to apply a 3 V upper limit for *E*_d_, and a −3.5 V upper limit for *E*_c_ (that is, we look for materials with *E*_d_<3 V and −*E*_c_>3.5 V). A more negative *E*_c_ implies a coating more stable upon charging. In addition, we also eliminate materials that contain radioactive elements and/or relatively rare elements, using production and reserve Herfindahl–Hirschman index (HHI) <9,000 as a proxy for availability^75^.

We consider two strategies to rank materials for each of the physical barrier, HF-barrier, and HF-scavenger cases in Table 1. In the first strategy, we use a global weighted-sum objective function *F*(*x*) as,


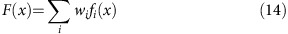


where *w*_*i*_ and *f*_*i*_ denote the weight (or importance) and the scaled value of attribute *i* for a given coating candidate *x*, respectively^76^. For each coating category we have a different set of scaled attributes, *f*_*i*_, considered in *F*. For physical barrier, HF-barrier and HF-scavenger coatings, these attribute sets are {*E*_c_, *E*_d_}, {*E*_c_, *E*_d_, *G*_s−HF_} and {*E*_c_, *E*_d_, *E*_d_(products), *G*_s−HF_}, respectively. This method maps the MOOP onto a single global objective *F*, which gives an overall ‘performance' score for a coating candidate which can be used to rank the materials. Weighted-sum method, however, requires several critical assumptions to be made. First, *w*_*i*_ -the relative importance of an attribute- has to be defined *a priori*, which often requires higher-level information about the problem other than just the attributes themselves^76^. Second, the final value of *F*(*x*) is not invariant under scaling or transformation of attribute data with different methods. Distribution of scaled attributes are not necessarily similar either, that is, they may be skewed, resulting in an inherent bias (intrinsic weights) in *F*(*x*) (Supplementary Fig. 4). For our weighted-sum analysis, we assume equal *w*_*i*_, and use a min-max normalization to scale the original (unscaled) 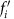 to a range of [0, 1], that is, *f*_*i*_(*x*)=(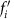
(*x*)−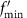)/(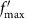
−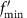). For *f*_*i*_(*x*), we assume that 0 and 1 correspond to the ‘worst' and the ‘best' extrema of attribute *i* in the data set, respectively.

Due to the above-mentioned assumptions and pretreatments required in the weighted-sum approach, we implement rank aggregation^77^ as an alternative materials selection method that does not require data scaling or transformation. In this approach, we first generate separate lists of material rankings for each attribute *i*, and then find a super-list that is as close as possible to all these individual lists. To measure the distance between two lists *a* and *b*, we use Spearman's footrule distance defined as *d*(*a*, *b*)=∑_*x*_ |
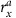
−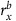
| where 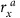 is the rank of material *x* in list *a*. A brute-force approach to rank aggregation becomes intractable even for small lists of about 10 to 15 candidates, and therefore we used a cross-entropy Monte Carlo method available in the RankAggreg package by Pihur *et al*.^77^

### Data availability

Attributes calculated for each material considered in this work and the extended list of weighted-sum materials rankings are provided as Supplementary Data 1 and Supplementary Data 2, respectively. All other relevant data are available from the authors.

## Additional information

**How to cite this article:** Aykol, M. *et al*. High-throughput computational design of cathode coatings for Li-ion batteries. *Nat. Commun.* **7,** 13779 doi: 10.1038/ncomms13779 (2016).

**Publisher's note**: Springer Nature remains neutral with regard to jurisdictional claims in published maps and institutional affiliations.

## Supplementary Material

Supplementary Information Supplementary Figure, Supplementary Table and Supplementary Reference

Supplementary Data 1 Calculated material attributes of coating candidates.

Supplementary Data 2 Extended list of weighted-sum rankings of coating materials.

## Figures and Tables

**Table 1:** Coating functionalities and corresponding attributes used in the HT coating design framework.

Functionality	Thermodynamic stability	Electrochemical stability	HF reactivity
Physical barrier	High	High	—
HF-scavenger	High	High	High
HF-barrier	High	High	Low

HF, hydrofluoric acid.

**Table 2:** Optimal and nearly-optimal coatings for LiCoO_2_ and LiMn_2_O_4_.

Cathode	Optimal coatings	Nearly optimal coatings
LiCoO_2_	Li_2_SrSiO_4_, Li_2_CaSiO_4_, CaIn_2_O_4_, SrHClO, SrBrOH, SrHfO_3_	Li_5_ReO_6_, Sr_2_MgWO_6_, Li_4_H_3_BrO_3_, Li_4_H_3_ClO_3_, Sr_2_LiReO_6_, Sr_2_CaWO_6_, SrZrO_3_
LiMn_2_O_4_	Li_2_GeO_3_, Sr_2_Nb_2_O_7_, Pb_3_Cl_2_O_2_, Pb_3_Br_2_O_2_, Li_2_TiGeO_5_, Li_2_TiSiO_5_, Li_4_NiTeO_6_, Ca_2_Mn_3_O_8_, Li_2_MnO_3_, Pb_2_SO_2_, PbHClO, PbBrOH, Ba_2_Hg_3_Pd_7_O_14_	CaTa_2_O_6_, Pb_3_O_4_, SrBrOH, Ba_2_TiSi_2_O_8_, Ba_2_Ti_4_Fe_2_O_14_, Sr_2_TaFeO_6_, SrPd_3_O_4_, SrHClO, Sr_2_NbFeO_6_, CaPd_3_O_4_

Nearly-optimal coatings correspond to the relaxed *E*_c_ filter as explained in the text.
