# Supplementary material for: High-throughput computational design of cathode coatings for Li-ion batteries
Source: Nat Commun. 2016 Dec 14;7:13779. doi: 10.1038/ncomms13779 (PMC5171834; doi:10.1038/ncomms13779)
Supplement: Supplementary Information — Supplementary Figure, Supplementary Table and Supplementary Reference [file ncomms13779-s1.pdf]

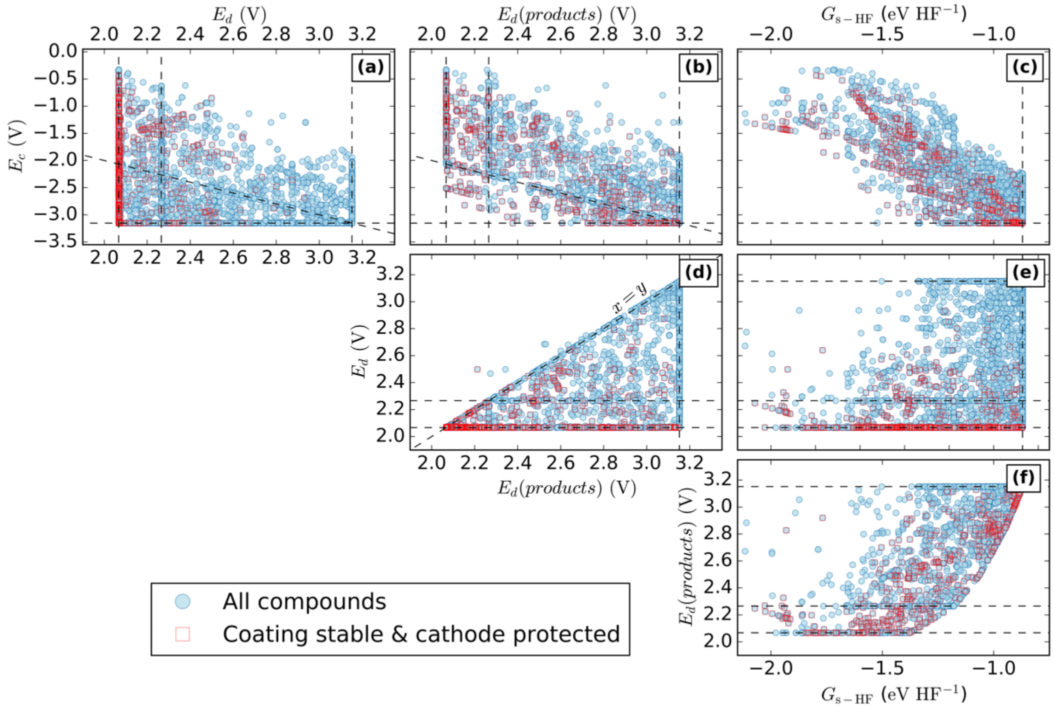

Supplementary Fig. 1. Coating attribute matrix in presence of $LiCoO_2$. Each subplot from (a) to (f) in the matrix plot compares pairs of attributes when $LiCoO_2$ is included in the chemical space.

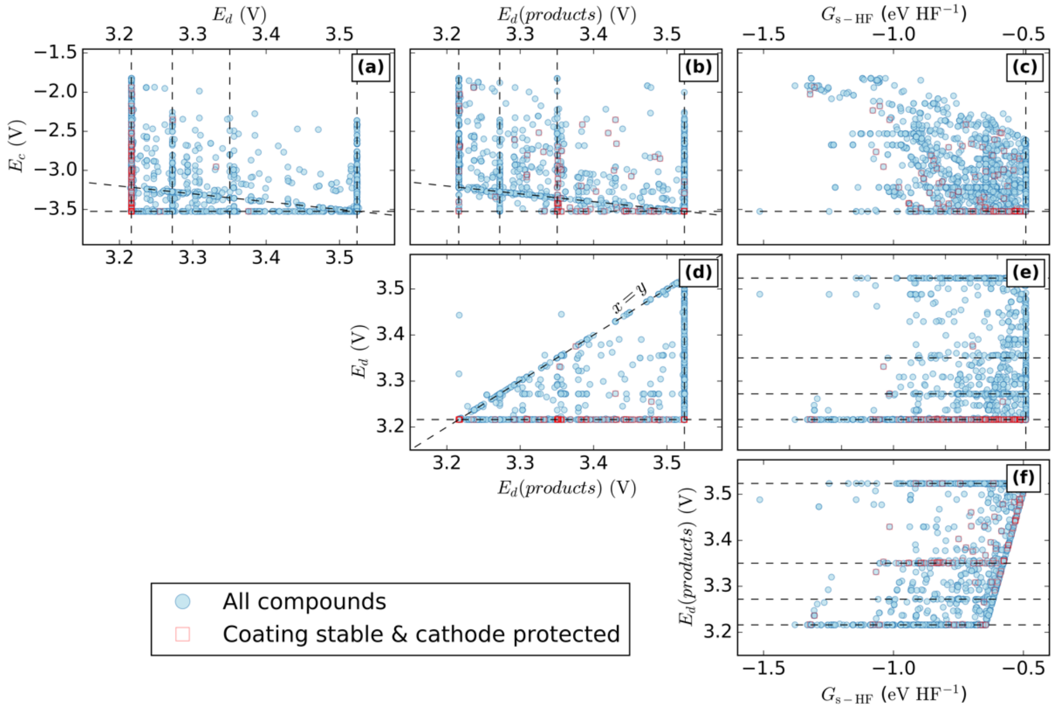

Supplementary Fig. 2. Coating attribute matrix in presence of $LiMn_2O_4$. Each subplot from (a) to (f) in the matrix plot compares pairs of attributes when $LiMn_2O_4$ is included in the chemical space.

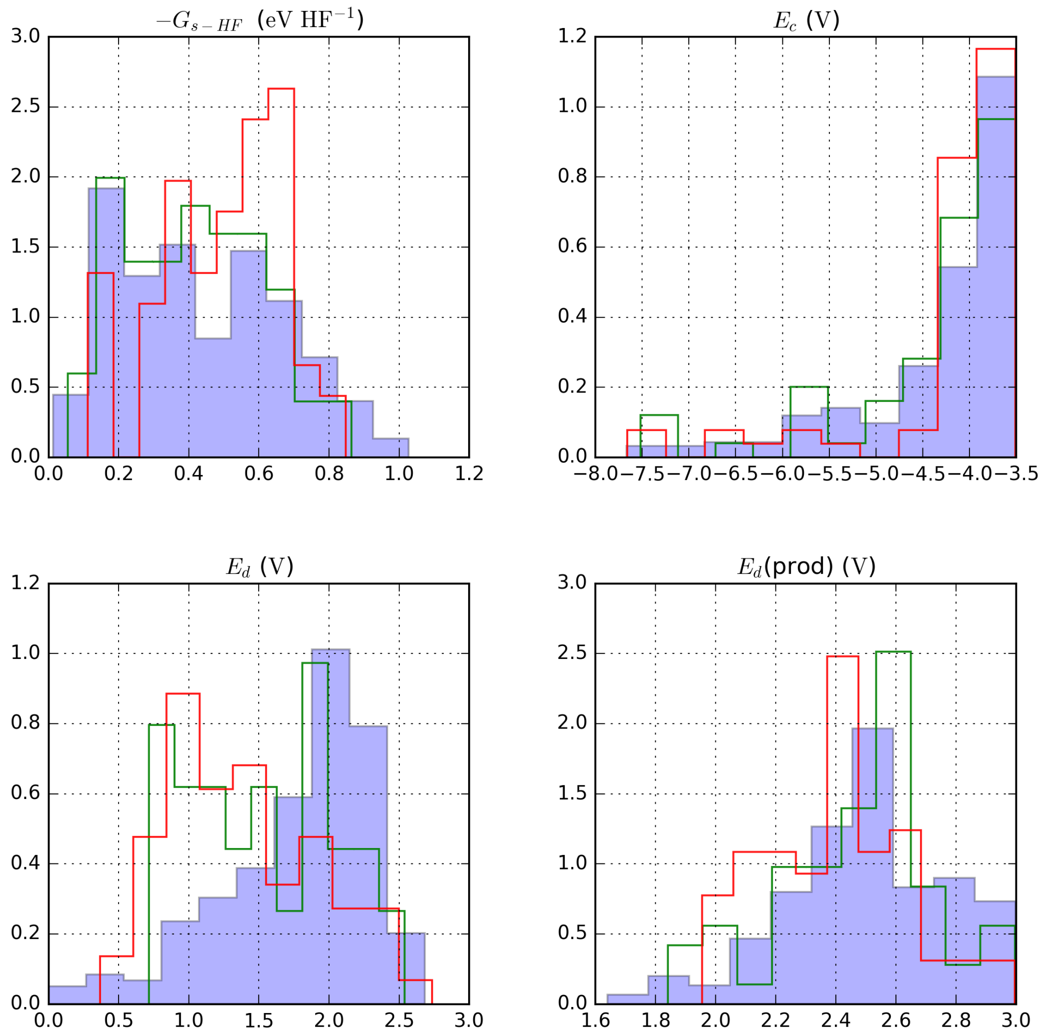

Supplementary Fig. 3. Histograms of design attributes for oxides, borates and silicates in MOOP framework. The distribution of thermodynamic attributes of HF-scavenger borates and silicates are similar to that of oxides, except for the stability against reduction, where borates and silicates yield more low voltage candidates (i.e. stable) compared to oxides. As all other attributes are similar, the additional electrochemical stability makes borates and silicates more effective candidates in our framework.

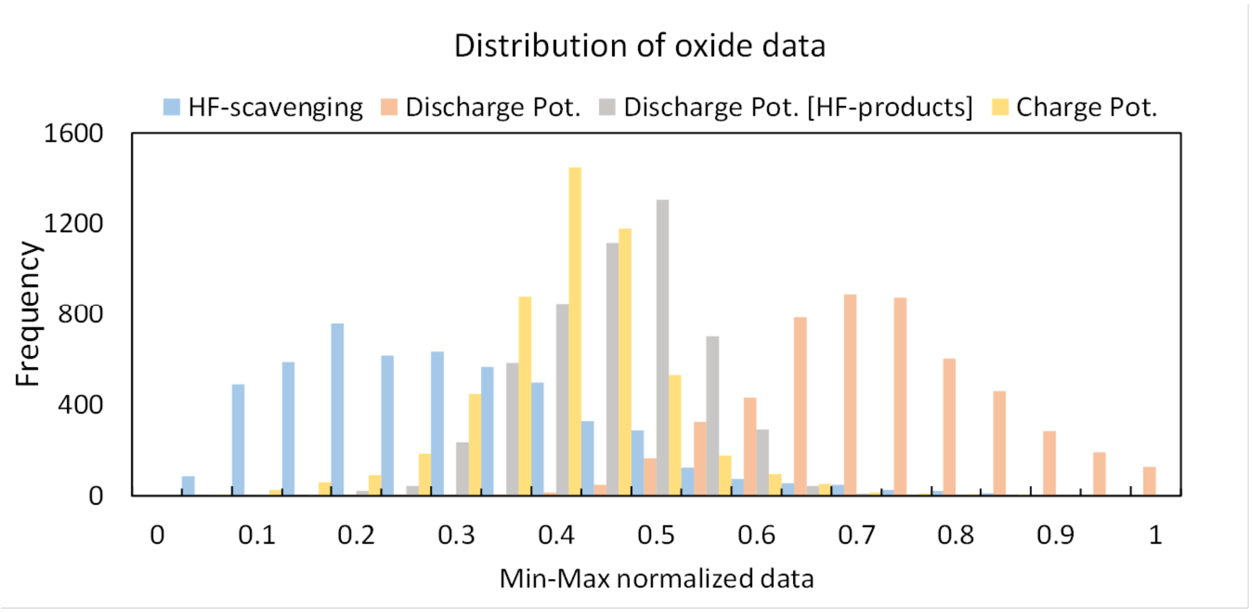

Supplementary Fig. 4. Distribution of normalized attributes used in the design framework. These attributes are charge and discharge potentials (including discharge potential of HF-scavenging reaction products) and HF-scavenging reaction energies. It is clearly observed that shapes of distributions, including the means vary among attributes. For example, charge potential is emphasized more than the other attributes in the global-objective function $F(x)$ since it skewed more towards higher $f(x)$ values.

Supplementary Table 1. Systematic reactions observed for selected cathode materials. These are found automatically within the framework based on the bulk materials available in the OQMD, and therefore do not necessarily represented the actual insertion/extraction reactions, but provide approximate limits obtained from bulk thermodynamics.

**$LiCoO_2$**	
Electrochemical reaction	E (V)
$3LiCoO_2 + 2Li \rightarrow Li_5CoO_4 + 2CoO$	2.07
$Co_3O_4 + Li \rightarrow LiCoO_2 + 2CoO$	2.27
$Co_3O_4 + 2\ Li_2CoO_3 + Li \rightarrow 5LiCoO_2$	3.15
HF-attack reaction	$\Delta G_{\mathrm{s-HF}}$ (eV HF^{-1})
$4LiCoO_2 + 2HF \rightarrow Co_3O_4 + 2LiF + Li_2CoO_3 + H_2O$	-0.87

**$LiMn_2O_4$**	
Electrochemical reaction	E (V)
$2MnO_2 + Li \rightarrow LiMn_2O_4$	3.52
$3LiMn_2O_4 + 2H_2O + Li \rightarrow Li_2MnO_3 + 4MnOOH$	3.35
$Mn_5O_8 + Li \rightarrow Mn_3O_4 + LiMn_2O_4$	3.27
$5LiMn_2O_4 + 3Li \rightarrow 4Li_2MnO_3 + 2Mn_3O_4$	2.22
HF-attack reaction	$\Delta G_{\mathrm{s-HF}}$ (eV HF^{-1})
$LiMn_2O_4 + HF \rightarrow MnO_2 + LiF + MnOOH$	-0.49

Supplementary Table 2. Experimental data used in the framework. The listed data is employed for gaseous reference states, dilute-limit HF in electrolyte and liquid H_2O in our framework along with the OQMD free energies and standard electrode potentials.[1,2] Entropies of gases are used to approximate the room temperature free energy of formation of compounds using the 0 K DFT data from the OQMD as explained in the manuscript. To the $\Delta_f G^0$(HF) listed below, we further add an activity contribution ($298R \times \ln[activity]$) assuming an HF concentration of 10^{-6} for completeness (where R is the gas constant). However, it is worth noting that the absolute value of the free energy of HF is only a constant in all HF-scavenging reactions, and therefore does not alter the relative rankings of coatings with respect to reaction energies. Water, on the other hand, competes with the OQMD phases that contain H and O, upon determining the HF-scavenging reaction products. Therefore to reasonably approximate $\Delta_f G^0$(H_2O, liquid) while ensuring consistency with the rest of the OQMD calculations, we add the experimental enthalpy of melting (which is relatively small at ~0.02 eV atom^{-1}, compared to entropy contribution) and the room temperature entropy contribution ($-\mathrm{T} \times \mathrm{S_{liq}}$ listed below) to the OQMD free energy of the stable H_2O polymorph in the OQMD.

Species	$298 \times S^{0,exp}_{298K}$ (eV atom^{-1})	$\Delta_f G^{0.exp}_{298K}$ (eV atom^{-1})
O_2 (g)	0.317	
F_2 (g)	0.313	
Cl_2 (g)	0.345	
H_2 (g)	0.202	
N_2 (g)	0.296	
HF, aqueous (infinitely dilute)		-1.444
H_2O (liq)	0.072	

Supplementary References

1. Chase, M. W. *et al.* JANAF Thermochemical Tables Third Edition. *J. Phys. Chem. Ref. Data.* **14,** Suppl. 1 (1985).
2. Wagman, D. D. *et al.* The NBS Tables of Chemical of Chemical Thermodynamic Properties. *J. Phys. Chem. Ref. Data* **Vol. 11,** Suppl. 2 (1982).
